# A prospective cohort study linking migration, climate, and malaria risk in the Peruvian Amazon

**DOI:** 10.1017/S0950268823001838

**Published:** 2023-11-30

**Authors:** Annika K. Gunderson, Cristina Recalde-Coronel, Benjamin F. Zaitchick, Pablo Peñataro Yori, Silvia Rengifo Pinedo, Maribel Paredes Olortegui, Margaret Kosek, Joseph M. Vinetz, William K. Pan

**Affiliations:** 1 Department of Epidemiology, Gilling School of Global Public Health, University of North Carolina at Chapel Hill, Chapel Hill, NC, USA; 2 Duke Global Health Institute, Duke University, Durham, NC, USA; 3 Department of Earth and Planetary Sciences, Johns Hopkins University, Baltimore, MD, USA; 4 Facultad de Ingeniería Marítima y Ciencias del Mar, Escuela Superior Politécnica del Litoral, Guayaquil, Ecuador; 5 Asociación Benéfica Prisma, Iquitos, Peru; 6 Division of Infectious Diseases, University of Virginia, Charlottesville, Virginia, USA; 7 Section of Infectious Diseases, Department of Internal Medicine, School of Medicine, Yale University, New Haven, USA; 8 International Centers of Excellence for Malaria Research – Amazonia, Laboratorio de Investigación y Desarrollo, Facultad de Ciencias y Filosofía, Universidad Peruana Cayetano Heredia, Lima, Peru; 9 Laboratorios de Investigación y Desarrollo, Facultad de Ciencias y Filosofía, Universidad Peruana Cayetano Heredia, Lima, Peru; 10 VA Connecticut Healthcare System, West Haven, CT, USA; 11 Institute of Tropical Medicine Alexander von Humboldt, Universidad Peruana Cayetano Heredia, Lima, Peru; 12 Nicholas School of the Environment, Duke University, Durham, NC, USA

**Keywords:** malaria, migration, *Plasmodium falciparum*, *Plasmodium vivax*, Peru Amazon

## Abstract

Migration is an important risk factor for malaria transmission for malaria transmission, creating networks that connect *Plasmodium* between communities. This study aims to understand the timing of why people in the Peruvian Amazon migrated and how characteristics of these migrants are associated with malaria risk. A cohort of 2,202 participants was followed for three years (July 2006 - October 2009), with thrice-weekly active surveillance to record infection and recent travel, which included travel destination(s) and duration away. Migration occurred more frequently in the dry season, but the 7-day rolling mean (7DRM) streamflow was positively correlated with migration events (OR 1.25 (95% CI: 1.138, 1.368)). High-frequency and low-frequency migrant populations reported 9.7 (IRR 7.59 (95% CI:.381, 13.160)) and 4.1 (IRR 2.89 (95% CI: 1.636, 5.099)) times more *P. vivax* cases than those considered non-migrants and 30.7 (IRR 32.42 (95% CI: 7.977, 131.765)) and 7.4 (IRR 7.44 (95% CI: 1.783, 31.066)) times more *P. falciparum* cases, respectively. High-frequency migrants employed in manual labour within their community were at 2.45 (95% CI: 1.113, 5.416) times higher risk than non-employed low-frequency migrants. This study confirms the importance of migration for malaria risk as well as factors increasing risk among the migratory community, including, sex, occupation, and educational status.

## Introduction

Deforestation and seasonal climate anomalies are thought to contribute to malaria transmission risk, often conceptualised through impacts on local vector populations [1, 2]. An important, yet understudied, aspect is how environmental conditions influence malaria propagation through demographic risk behaviours, particularly migration. Migration is defined as travel across administrative boundaries, including international and internal travel for permanent relocation, or circular migration, which is temporary travel from an individual’s home to another location with the intention of returning to their home community [3, 4]. Although circular migration has been identified as an important factor in malaria transmission [5], empirical research linking migration and malaria focuses primarily on large-scale migration events, permanent relocation, and international movement [6–9].

Migration patterns are underpinned by the human ecology of movement. These 7apatterns are determined by the individual family based on their particular needs, revolving around a set of characteristics of the family, or even a community including but not limited to having dependents and economic opportunities [10]. In the Peruvian Amazon, demographic, political-economic, socio-economic, and ecological factors play a role in the decision for migration [10, 11]. Access to education and higher incomes may entice movement between communities, whereas poor crop yields and lack of employment opportunities or markets may force people to leave their communities [12–14]. Ecological factors revolving around agriculture and climate change are particularly important to the Amazonian context as the main occupations in these communities remain agriculture, fishing, and logging. Each of these occupations requires travel to work sites, which may depend on rainfall and river height as most of this travel occurs along the river system as roads are rarely available. Such movement may increase exposure to mosquitoes, of which vector reproduction rates and human–vector interactions have increased in part due to climate change and migration [15–17].

A framework for linking migration patterns with vector-borne diseases was recently proposed to describe how movement frequency and distance are key components to understanding transmission of vector-borne diseases [18]. While this framework has been applied to studies of malaria transmission [19], the key components missing that inform malaria control are the underlying drivers of migration, namely, social and demographic and seasonal/climate factors that influence migration decisions.

While migration itself has been recognised as a strong risk factor for malaria infection, migrant populations are heterogeneous, requiring more than a simple bifurcation of migrants vs. non-migrants [20–24]. Differentiating migrants by key characteristics of travel patterns (duration and destination of travel) and social and demographic factors (education level, occupation, and socio-economic status (SES)) identifies specific aspects of migrant populations that increase their risk of disease beyond the initial divisions of separating migrants from non-migrants [20, 25]. Characterising these subpopulations and their proclivities for seasonal travel increases our understanding of risk distribution among the migrant population as a whole [26].

This study aimed to test the hypothesis that river height plays a role in the decision to travel and that high-frequency migrants of lower SES will be at increased risk of contracting either *Plasmodium vivax* or *Plasmodium falciparum* than non-migrants or low-frequency migrants of higher SES. We explored potential drivers of migration within the rural Amazon context and how such determinants and migration events related to malaria risk. Specifically, this study evaluated [1] the social and climactic factors associated with the timing of migration events through seasonal migration streams, and [2] migration typologies associated with risk for contracting symptomatic or asymptomatic malaria.

## Methods

### Study area and population characteristics

**Figure 1.**
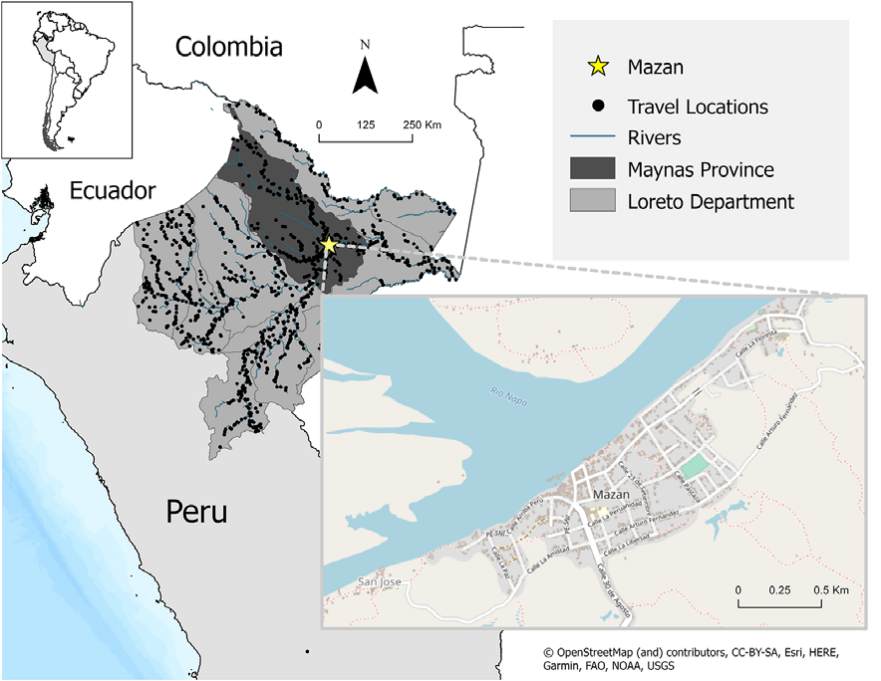
Map of the study area Top left corner shows the location of Peru in relation to other countries in South America. Map of Peru in the left indicated Loreto by the intermediate gray coloration and the province of Maynas in dark gray. The province of Maynas is where Mazan is located, as seen by the yellow star. The black dots across Loreto represent a sample of the locations study participants visited during the study period. The map on the right is an enlargement of the community of Mazan. San Jose and Puerto Alegre are smaller communities located less than 10 km from Mazan in Maynas province. Though not depicted in the enlargement above, both communities fall may also be represented by the yellow star in the map of Loreto.

This analysis represents a secondary analysis of data originally collected from participants recruited from July 2006 to August 2009 as part of a prospective, population-based malaria study in the Mazan district of Maynas Province in Loreto, Peru (Figure 1). The population is primarily mestizo who live approximately 2 h by boat from the city of Iquitos. Temperatures are relatively stable, with diurnal temperatures fluctuating between 23.3 and 31.6 °C, with less than 0.5% of days falling below the minimum temperature for anopheles’ reproduction, the vector species that transmits malaria. Precipitation is the highest from February to July with river heights highest in April and May. The population is highly mobile, travelling along rivers for economic and leisure activities, including logging, fishing, farming, and visiting relatives.

### Data collection

Participants were selected following a census in the communities of Mazan, San Jose, and Puerto Alegre: All households in San Jose and Puerto Alegre were invited to participate, while half (every other house) in Mazan were invited (Figure 1). Everyone living in a selected house was eligible to enrol, including those joining the household after initial enrolment (i.e. newborns or individuals permanently moved into the home). Short-term visitors were not eligible. A total of 2,422 participants from 402 families were enrolled, 237 refused, and 430 were lost to follow-up due to death, permanent relocation outside the study area, or refusal after enrolment. Enrolment surveys were administered to collect household demographic and economic information, and assets. Participants were visited thrice weekly for symptomatic screening and recording recent travel history (destination and duration). Blood samples via finger prick were taken for microscopic examination (thick and thin blood films) and PCR testing (dried blood spot using Whatman filter paper) each time participants reported travel farther than 10 km from their home, trips longer than 72 h, or symptoms associated with malaria infection, including fever, headache, and chills. Trips to Iquitos, the largest city in Loreto, were excluded because there is no known stable malaria transmission within the city. Blood samples were also collected on enrolment and quarterly thereafter.

### Laboratory testing

Thick and thin blood smears were examined for *Plasmodium* using light microscopy at a local Ministry of Health post. Positive samples were verified using specific nested PCR for *P. vivax*, *P. falciparum*, *P. malariae*, and *P. ovale.* Samples with *P. vivax* underwent PCR-RFLP analysis on the PvMSP-3α locus using the AluI and HhaI restriction enzymes as stipulated in Bruce et al. [27].

### Hydrometeorological data

Daily streamflow, temperature, and rainfall data were extracted from the Malaria Early Warning System Land Data Assimilation System (MEWS-LDAS) [28]. MEWS-LDAS includes Climate Hazard Group InfraRed with Stations precipitation estimates [29]and downscaled Global Data Assimilation System meteorological fields, and generates streamflow estimates using the Noah-MultiParameterization Land Surface Model [30] in combination with the Hydrological Modeling and Analysis Platform (HyMAP) routing model [31], implemented with the NASA Land Information System. [32] Streamflow is a measure of how much water is flowing in a river at any instant (m^3^/s) for a defined cross-section, which we identified as a section of the Napo River adjacent to the study site (area of interest: latitude = −3.60, latitude = −3.40, longitude = −73.20, longitude = −73.00). Daily hydrometeorological data were merged with participant migration status; however, temperature and rainfall were excluded from analyses due to multicollinearity.

### Outcomes

The primary outcome for this study was the cumulative incidence of malaria (by species) across the study period. Malaria was defined as symptomatic or asymptomatic infection detected from a positive blood smear. An infection occurring within 28 days of a prior case was considered a recurrence of the earlier infection and excluded [33]. Participants were assumed to not have malaria with the absence of a positive malaria diagnosis and were allowed to record more than one malaria infection throughout the study.

The timing of migration decisions was a secondary outcome in this study. Migration timing is the date of departure for any short-term, temporary travel lasting longer than 2 days within or outside of Peru, including circular migration. Migration timing was assessed on a daily scale, matching hydrometeorological data.

### Exposure parameters

**Figure 2.**
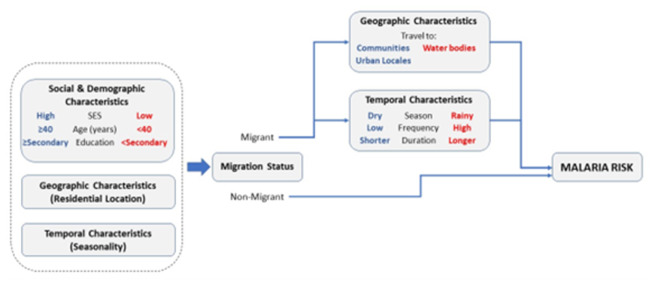
The red and blue highlighted text indicate hypotheses of the relationship between the variable and malaria risk. Red indicates higher malaria risk while blue indicates lower. For example, high SES is hypothesised to be associated with lower malaria risk and low SES with higher risk.

Malaria incidence was evaluated against migration, sex, age, occupation, education, and socio-economic status as an index [34]. Migration was parameterised to capture a typology of risk (Figure 2). Firstly, individuals were categorised as being a migrant if they have travelled on average one or more times per year irrespective of season and a non-migrant if they travelled less than once per year. Secondly, migrants were classified as low- vs. high-frequency migrants if they travelled between an average of one and three times a year vs. four or more times a year during the study, respectively. The cutoff separating low- vs. high-frequency migrants was defined using the average annual number of trips taken by the remining participants (2.85). Thirdly, using a migration typology framework (Figure 2) [20], we defined migrant characteristics to evaluate malaria risk in migrant subpopulations. We defined social and demographic characteristics of migrants that include occupation, sex, age, education level, and the number of individuals living in a household, each measured at enrolment. In addition, we evaluate malaria risk based on migration destination type (community, river, lake, seasonal waterway), direction of travel (urban to rural or rural to urban), seasonality, and travel duration (days).

A socio-economic status index was created that measures access to water and sanitation, assets, maternal education, and income (WAMI) for each participant, which has been used previously in Peru [34]. The average number of years of schooling for the head of the household and their spouse was used as a proxy for maternal education.

To evaluate the factors associated with the timing of migration events, we assessed streamflow as a 7-day rolling average preceding each surveillance day. This time interval was determined through a sensitivity analysis including 14-, 21-, and 28-day ranges of streamflow measures.

### Statistical analysis

Descriptive data of cohort participants were tabulated for the variables included in the migration typology. Study participants were included in this analysis if they contributed 6 or more months of person-time to ensure a minimum of three malaria tests, resulting in 220 participants being omitted (note: there were no significant differences between participants omitted and retained for migration typology or malaria incidence). The time individuals spent away travelling was not included in a participant’s person-time.

Separate logistic regression models with household random effects were run to evaluate streamflow and seasonality factors associated with migration. As noted previously, 7-day lagged rolling means (7DRMs) and differences of streamflow for 1, 2, 3, and 4 weeks before participant departure were evaluated in a sensitivity analysis. AIC values were compared to determine the covariates to include during model selection from season, sex, age, occupation, education level, and travel frequency.

Separate negative binomial models with household random effects were fit to evaluate factors associated with combined symptomatic and asymptomatic cumulative malaria incidence over the study period, by *Plasmodium* species. Models were adjusted for known risk factors of malaria, including sex, occupation, age, education level, income, marital status, and SES. Malaria models were further stratified by age (under 15 years vs. 15 years and older) to evaluate the effect of low- vs. high-frequency migrants. These models included interaction terms for migration status and variables of the migration typology to understand their combined effect on malaria risk. Model results were reported as incidence rates and incidence rate ratios with 95% confidence intervals.

Variable selection in both the migration and malaria models was conducted by first evaluating potential collinearity among independent variables, then using backward stepwise regression, omitting variables with p > 0.15. Interaction terms and model fit were assessed using the likelihood ratio test and comparing Akaike information criterion (AIC) values. Data analysis was conducted in STATA SE 16 (64-bit) from September 2020 to March 2021.

### Human research ethics

All participants who were 18 years and older completed written consent forms. Parental permission and informed assent were obtained for children aged 12–17 years and younger than 12 years. IRB approval was provided by the University of California San Diego (FWA00004495), Universidad Peruana Cayetano Heredia (FWA00000525), the Johns Hopkins Bloomberg School of Public Health (FWA00000287), and Asociación Benéfica Prisma (AB Prisma) (FWA00001219). Data are available upon request to Dr. Joe Vinetz (joseph.vinetz@yale.edu).

## Results

### Characteristics of the study population

**Figure 3.**
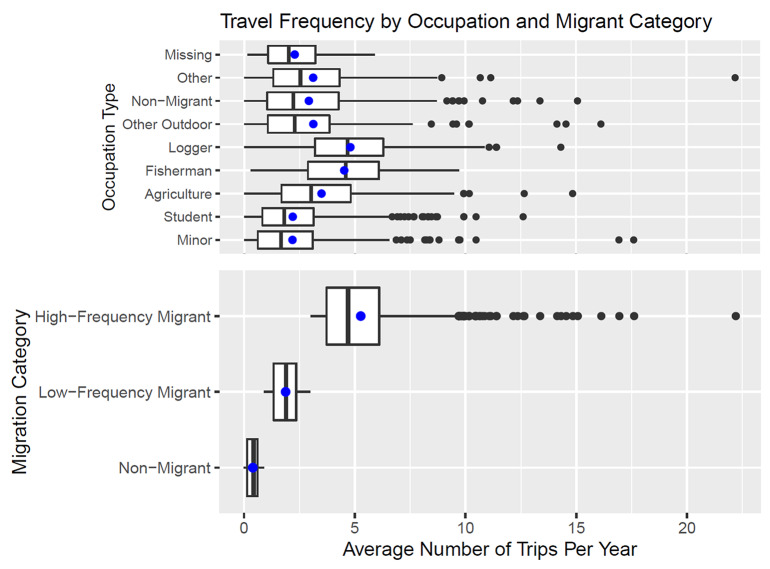
Travel frequency by occupation and migrant category. Estimates are the average number of trips taken by all members of a given occupation or migrant category over one year. Non-migrant occupations included here are motorbike drivers, craftsmen, and local businessmen. This category is used to classify occupation and is different from the non-migrant migration status category. Blue circles within the box plot indicate the average in contrast to the boxes, indicating the interquartile range and median.

Between July 2006 and October 2009, 2,202 individuals from 402 families were enrolled into the study. Of these participants, 504 individuals (22.9%) were classified as non-migrants, 850 (38.6%) as low-frequency migrants, and 848 (38.5%) as high-frequency migrants (Table 1). High-frequency migrants were more likely to be male (63.4%), of older age (median age 23 (IQR: 12–37.5)) years for high-frequency migrants vs. 15 (IQR: 7–31) years and 11 (IQR: 2–25) years for low-frequency migrants and non-migrants, respectively, and be employed in logging, agriculture, and fishing occupations compared to low-frequency migrants and non-migrants. Occupational logging was particularly common among high-frequency migrants (20.6%) compared to low-frequency migrants (4.6%) and non-migrants (2.8%). High-frequency migrants reported a median of 5.5 trips per year, and low-frequency migrants reported taking a median of 2.5 trips a year (Figure 3). Loggers and fisherman travelled the most, with medians of 4.7 and 4.6 trips per year, respectively.

**Table 1.** Characteristics of the study population

	All participants *N* (%)	*P. vivax* ^a^ *N* (%)	*P. falciparum* ^a^ *N* (%)	Non-migrants*N* (%)	Low frequency migrants *N* (%)	High frequency migrants *N* (%)
	2,202 (100)	556	303	504 (22.9)	850 (38.6)	848 (38.5)
Sex (%)*						
Female	1,044 (47.4)	191 (34.4)	89 (29.4)	291 (57.7)	442 (52.0)	311 (36.7)
Male	1,158 (52.6)	365 (65.6)	214 (70.6)	213 (42.3)	408 (48.0)	537 (63.3)
Age (Continuous)						
Median (IQR)	17 (8–33)	21 (13–35)	25 (15–40)	11 (2–25)	15 (7–31)	23 (12–37.5)
Age (%)*						
0–9	627 (28.5)	89 (16.0)	30 (9.9)	217 (43.1)	259 (30.5)	151 (17.8)
10–14	347 (15.8)	77 (13.9)	39 (12.9)	88 (17.5)	157 (18.5)	102 (12.0)
15–19	227 (10.3)	85 (15.3)	44 (14.5)	32 (6.4)	85 (10.0)	110 (13.0)
20–29	348 (15.8)	117 (21.0)	66 (21.8)	58 (11.5)	121 (14.2)	169 (19.9)
30–39	245 (11.1)	77 (13.9)	47 (15.5)	40 (7.9)	83 (9.8)	122 (14.4)
40–49	189 (8.6)	51 (9.2)	40 (13.2)	31 (6.2)	65 (7.7)	93 (11.0)
>49	219 (9.9)	60 (10.8)	37 (12.2)	38 (7.5)	80 (9.4)	101 (11.9)
Family size (%)						
1–3	148 (6.7)	29 (5.2)	18 (5.9)	25 (5.0)	53 (6.2)	70 (8.3)
4–6	844 (38.3)	210 (37.8)	89 (29.4)	207 (41.1)	324 (38.1)	313 (36.9)
7–9	671 (30.5)	171 (30.8)	112 (37.0)	140 (27.8)	280 (32.9)	251 (29.6)
>9	539 (24.5)	146 (26.3)	84 (27.7)	132 (26.2)	193 (22.7)	214 (25.2)
Education level (If older than 15) (%)						
None (<1 year)	75 (6.1)	24 (4.3)	18 (7.7)	16 (8.0)	22 (5.1)	37 (6.2)
Primary (1–6 years)	598 (48.7)	206 (20.7)	138 (59.0)	90 (45.2)	221 (50.9)	287 (48.2)
Secondary (7–11 years)	465 (37.9)	145 (14.6)	71 (30.3)	80 (40.2)	159 (36.6)	226 (38.0)
>Secondary (>11 years)	90 (7.3)	15 (1.5)	7 (3.0)	13 (6.5)	32 (7.4)	45 (7.6)
Socio-economic Status (%)**						
Q1	423 (19.2)	112 (20.1)	70 (24.4)	104 (20.6)	167 (19.7)	152 (17.9)
Q2	427 (19.4)	115 (20.7)	66 (23.0)	83 (16.5)	175 (20.6)	169 (19.9)
Q3	431 (19.6)	104 (18.7)	50 (17.4)	102 (20.2)	177 (20.8)	152 (17.9)
Q4	437 (19.8)	148 (26.6)	78 (27.2)	85 (16.9)	161 (18.9)	191 (22.5)
Q5	401 (18.2)	56 (10.1)	23 (8.0)	117 (23.2)	135 (15.9)	149 (17.6)
Unknown	83 (3.8)	21 (3.8)	0 (0)	13 (2.6)	35 (4.12)	35 (4.1)
Occupation (%)*						
Minor (>15)	401 (18.2)	57 (10.3)	20 (6.6)	139 (27.6)	158 (18.6)	104 (12.3)
Student	646 (29.3)	126 (22.7)	53 (17.6)	177 (35.1)	290 (34.1)	179 (21.1)
Agriculture	189 (8.6)	72 (13.0)	52 (17.3)	23 (4.6)	71 (8.4)	95 (11.2)
Fisherman	23 (1.0)	12 (2.2)	8 (2.7)	2 (0.4)	4 (0.5)	17 (2.0)
Logger	228 (10.4)	116 (20.9)	85 (28.2)	14 (2.8)	39 (4.6)	175 (20.6)
Other outdoor	108 (4.9)	23 (4.1)	6 (2.0)	23 (4.6)	44 (5.2)	41 (4.8)
Non-migrant work	396 (18.0)	90 (16.2)	48 (16.0)	88 (17.5)	162 (19.1)	146 (17.2)
Other/Unknown	211 (9.6)	60 (10.8)	29 (9.6)	38 (7.5)	82 (9.7)	91 (10.7)

*Note*: Description of the study population divided into migration frequency categories. *N* is the number of individuals in each group, using column percentages. Significant differences between characteristic distribution across migrant frequency categories designated by *P < 0.001; **P < 0.05.

a Counts of positive cases of *P. vivax* and *P. falciparum* among the entire cohort. Participants with sperate infections from both *Plasmodium* species are included in both columns.

### Travel and hydrometeorology

The 7 DRM streamflow ranged from 3,400 to 7,120 m^3^/s, with an annual average of 4,621 m^3^/s. The average 7 DRM streamflow when individuals did not travel was 4615m^3^/s compared to 4,633 m^3^/s on days when study participants did travel (Supplementary Figure S1). Higher 7 DRM streamflow occurred during the rainy season (February through July) at 5154 m^3^/s (ranging from 3,400 to 7,120 m^3^/s) compared to a 7 DRM 4142 m^3^/s in the dry season (ranging from 3,435 to 4,773 m^3^/s). Crude estimates of travel events indicate just over half of the recorded travel events occurred in the dry season (August to January), accounting for 126,019 (51.1%) trips taken during the study period, while 120,402 (48.9%) trips were taken in the rainy season. However, the dry season was negatively associated with migration, with 0.93 (95% CI: 0.905, 0.958) times the odds of travel in the dry vs. rainy season when adjusting for age, sex, and travel frequency of individuals (Table 2). Males were more likely to travel than women regardless of the month or season and had 1.700 (95% CI: 1.556, 1.858) times the odds of travelling during the rainy season as compared to the dry season (Table 2).

**Table 2.** Logistic regression results for climate factors and travel departure

	Model using the natural log 7-day rolling mean streamflow	Model using season
	OR (95% CI)	OR (95% CI)
Intercept	0.001 (0.000, 0.001)	0.003 (0.003, 0.004)
7-Day rolling mean streamflow	1.247 (1.138, 1.367)	---
Seasonal travel frequency	1.043 (1.038, 1.047)	1.044 (1.040, 1.049)
Season		
Rainy (February–July)	---	Reference
Dry (August–January)	---	0.931 (0.905, 0.958)
Sex		
Female	Reference	Reference
Male	1.207 (1.019, 1.430)	1.700 (1.556, 1.858)
Age (years)		
0–9	Reference	Reference
10–14	1.344 (1.015, 1.780)	1.257 (1.091, 1.448)
15–19	1.285 (0.935, 1.765)	2.396 (2.041, 2.813)
20–29	1.995 (1.515, 2.627)	2.264 (1.971, 2.600)
30–39	2.252 (1.653, 3.070)	2.093 (1.792, 2.444)
40–49	1.902 (1.355, 2.670)	2.172 (1.831, 2.576)
>49	1.063 (0.769, 1.468)	1.889 (1.605, 2.223)

*Note*: Odds ratios (ORs) and 95% confidence intervals for each of the three logistic regression models run comparing streamflow measures or season and departure dates. This is based on multivariate models using 1,819,818 observations.

“---” indicates that a value is not applicable to that model.

Model results indicate that elevated streamflow was associated with higher odds of travel, even after controlling for other covariates (sex, age, and average trips taken per season) (Table 2). A one unit increase in the natural log of the 7DRM streamflow was associated with 1.247 (95% CI: 1.138, 1.367) times in the odds of travel. Males were consistently more likely to travel than females in both models for streamflow and season. The age distribution of travel also varied between the two models with higher odds of travel occurring in all age groups for the dry vs. rainy season model, particularly for the 20–49 year age groups (Table 2). The effect of travel frequency was consistent in both models.

### Unadjusted estimates of Plasmodium vivax and P. falciparum risk association with migration typology

Cumulative malaria incidence rates did not vary by household size for either *P. vivax* or *P. falciparum* infections. Among non-migrants, only 5.8% (32/556) of individuals contracted *P. vivax* (Table 3). Based on the unadjusted rates, individuals in the high-frequency migration category reported having *P. vivax* 9.73 times as often as those considered non-migrant (95% CI: 1.85 to 18.01) cases of malaria per 1,000 person-years. Individuals employed in out-of-community manual labour, including fishing, logging, and agriculture, had a higher incidence of infection than those in any in-community work, with an IR of 21.61 (95% CI: 19.18, 24.36). Among non-migrants, 2.0% (6/303) contracted *P. falciparum* infection. Incidence rates increased for P*. falciparum* infections across migration categories (Table 3). High-frequency migrants reported 33.161 times as many cases as non-migrants per 1,000 person-years (95% CI: 8.156, 134.830). Participants considered to be more frequent travellers tend to register a higher number of malaria cases than those in lower migrant categories. Individuals employed in out-of-community manual labour, including fishing, logging, and agriculture, had a higher incidence of infection than those in any in-community work, with an IRR of 2.004 (95% CI: 1.060, 3.7768.21, 11.71).

**Table 3.** Bivariate incidence rates of *P. vivax* and *P. falciparum* across demographic typology characteristics (*N* = 2,202)

	*P. vivax*		*P. falciparum*	
	*N* (%)	IR (95% CI)	*N* (%)	IR (95% CI)
	556 (25.2)		303 (13.8)	
Migration status				
Non-migrant	32 (5.8)	1.85 (1.386, 2.471)	6 (2.0)	0.24 (0.108, 0.537)
Low-frequency migrant	178 (32.0)	7.52 (6.734, 8.393)	67 (22.1)	1.78 (1.418, 2.230)
High-frequency migrant	346 (62.2)	18.01 (16.768, 19.334)	230 (75.9)	7.39 (6.610, 8.256)
Sex				
Female	191 (34.4)	7.38 (6.678, 8.161)	89 (29.4)	2.03 (1.676, 2.457)
Male	365 (65.6)	12.88 (11.986, 13.845)	214 (70.6)	5.00 (4.456, 5.616)
Age (years)				
0–9	89 (16.0)	6.30 (5.476, 7.246)	30 (9.9)	1.09 (0.781, 1.529)
10–14	77 (13.8)	9.24 (7.914, 10.799)	39 (12.9)	3.14 (2.405, 4.099)
15–19	85 (15.3)	17.03 (14.782, 19.630)	44 (14.5)	5.17 (3.999, 6.691)
20–29	117 (21.0)	12.65 (11.079, 14.448)	66 (21.8)	4.82 (3.885, 5.973)
30–39	77 (13.8)	10.71 (9.016, 12.715)	47 (15.5)	5.11 (3.981, 6.549)
40–49	51 (9.2)	11.62 (9.623, 14.008)	40 (13.2)	5.33 (4.037, 7.027)
>49	60 (10.8)	10.90 (9.099, 13.053)	37 (12.2)	4.71 (3.580, 6.198)
Family size				
1–3	29 (5.2)	8.32 (6.476, 10.698)	18 (5.9)	3.14 (2.086, 4.723)
4–6	210 (37.8)	9.83 (8.923, 10.827)	89 (29.4)	2.58 (2.139, 3.119)
7–9	171 (30.8)	10.62 (9.565, 11.784)	112 (37.0)	4.48 (3.816, 5.262)
>9	146 (26.3)	11.08 (9,884, 12.413)	84 (27.7)	4.19 (3.483, 5.044)
Education level (if older than 15)^a^				
None (<1 year)	24 (6.4)	15.09 (11.615, 19.612)	18 (8.0)	7.82 (5.431, 11.247)
Primary (1–6 years)	198 (52.8)	13.466 (12.187, 14.878)	133 (59.1)	6.14 (5.296, 7.117)
Secondary (7–11 years)	138 (36.8)	12.14 (10.749, 13.700)	67 (29.8)	3.63 (2.905, 4.528)
>Secondary (>11 years)	15 (4.0)	4.72 (3.080, 7.246)	7 (3.1)	2.25 (1.211, 4.181)
Socio-economic status				
Q1	112 (20.1)	11.76 (10.361, 13.359)	70 (23.1)	4.55 (3.707, 5.579)
Q2	115 (20.7)	11.24 (9.906, 12.752)	66 (21.8)	4.10 (3.330, 5.058)
Q3	104 (18.7)	9.57 (8.346, 10.981)	50 (16.5)	3.00 (2.351, 3.837)
Q4	148 (26.6)	14.25 (12.710, 15.987)	78 (25.7)	4.54 (3.705, 5.563)
Q5	56 (10.1)	4.78 (3.942, 5.801)	23 (7.6)	1.49 (1.051, 2.101)
Occupation				
No work	183 (32.9)	7.42 (6.712, 8.196)	73 (24.1)	1.77 (1.445, 2.174)
Out-of-community manual labour	128 (23.0)	21.61 (19.180, 24.358)	93 (30.7)	9.80 (8.209, 11.706)
In-community manual labour	95 (17.1)	11.14 (9.556, 12.978)	58 (19.1)	5.57 (4.484, 6.914)
Not manual labour	148 (26.6)	10.29 (9.190, 11.520)	77 (25.4)	3.21 (2.625, 3.933)

*Note*: *N* represents the number of individuals in each group, using column percentages. Counts for positive cases of *P. vivax* and *P. falciparum* among the entire cohort. Participants with sperate infections from both *Plasmodium* species are included in both columns.

a Education level only includes participants older than 15 years old, as many participants below that age are not finished with their education.

The unadjusted incidence rate of *P. vivax* for individuals in the highest quintile for SES was 4.78 (95% CI: 3.94, 5.80) cases per 1,000 person-years. Similarly, those with a lower education level reported more infections, with an IR of 4.72 (95% CI: 3.08, 7.25). Males were at a higher risk of infection, with an IR of 12.88 (95% CI: 11.99, 13.84), than women, with an IR of 7.38 (95% CI: 6.68, 8.16).

Unadjusted incidence rates of *P. falciparum* for individuals in the highest quintile for SES were 1.49 (95% CI: 1.05, 2.10) cases per 1,000 person-years. Similarly, those with a secondary level or higher education reported less infections, with an IR of 4.72 (95% CI: 3.08, 7.25)). Males were at a higher risk of infection, with an IR of 5.00 (95% CI (4.46, 5.62), than women, with an IR of 2.03 (95% CI: 1.68, 2.46). Incidence rates stratified by migration status are given in Supplementary Tables S1 and S2 for *P. vivax* and *P. falciparum*, respectively.

Although more cases were reported by individuals who travelled to communities, unadjusted incidence rates for both *Plasmodium* species were greater for those who travelled to lakes, rivers, streams, and seasonal waterways. When comparing IR across destination type, streams, lakes, and rivers recorded 1.95, 2.36, and 1.81 times higher incidence rates for *P. vivax* than communities, respectively (Table 6). Significantly higher numbers of cases were associated with those who travelled to streams than those who did not travel to a stream at any point during the study. *P. vivax* infections were 10.667 times (95% CI: 8.860, 12.934) higher (Table 6).

The person-time contributed to travel to urban areas was considerably less than rural travel. Similarly, travel to communities and streams was greater than travel to lakes, rivers, and seasonal waterways. Trips last 1–7 days, and travel frequencies of 1–2 and 3–4 trips per season were the most common.

### Adjusted estimates for Plasmodium vivax

When controlling for sex, age, occupation, education level, SES, and marital status, low-frequency migrants were 2.93 (95% CI: 1.660, 5.170) and high-frequency migrants were 7.75 (95% CI: 4.473, 13.424) times as likely to contract *P. vivax* than non-migrants (Table 4). Individuals 20 years and older were at a reduced risk of malaria as compared to those aged 15–19 years. The incidence rate ratio (IRR) was 0.640 (95% CI: 0.456, 0.899) for study participants aged 20–29 years, 0.504 (95% CI: 0.345, 0.737) for those aged 30–39 years, 0.444 (95% CI: 0.296, 0.667) for those aged 40–49 years, and 0.391 (95% CI: 0.253, 0.605) for those older than 49 years (Table 3). Based on models including participants younger than 15 years, only IRRs for participants aged 10–14 years were significant at 1.763 (95% CI: 1.179, 2.368) compared to participants aged 0–4 years (Table 5).

**Table 4.** Negative binomial regression results for migration typology in participants over 15

	*P. vivax*		*P. falciparum*	
	IRR	95% Confidence Interval	IRR	95% Confidence Interval
Intercept	0.131	(0.053, 0.322)	2.556	(2.40e-13, 2.72e+13)
Migration status				
Non-migrant	Reference		Reference	
Low-frequency migrant	2.929	(1.660, 5.170)	7.486	(1.791, 31.288)
High-frequency migrant	7.749	(4.473, 13.424)	33.161	(8.156, 134.830)
Sex				
Female	Reference		Reference	
Male	1.430	(1.093, 1.872)	1.965	(1.387, 2.786)
Age (years)				
15–19	Reference		Reference	
20–29	0.640	(0.456, 0.899)	0.740	(0.497, 1.101)
30–39	0.504	(0.345, 0.737)	0.669	(0.420, 1.036)
40–49	0.444	(0.296, 0.667)	0.635	(0.402, 1.002)
>49	0.391	(0.253, 0.605)	0.505	(0.310, 0.823)
Education level				
No education	Reference		Reference	
Primary education	0.637	(0.402, 1.008)	0.627	(0.381, 1.032)
Secondary education	0.517	(0.313, 0.852)	0.391	(0.222, 0.690)
>Secondary education	0.316	(0.152, 0.657)	0.285	(0.118, 0.693)
SES				
Q1	Reference		Reference	
Q2	0.970	(0.682, 1.380)	0.828	(0.546, 1.254)
Q3	0.996	(0.697, 1.423)	0.745	(0.480, 1.157)
Q4	1.307	(0.922, 1.852)	0.892	(0.580, 1.372)
Q5	0.724	(0.473, 1.109)	0.365	(0.205, 0.649)
Occupation				
No work	Reference		Reference	
Out-of-community manual labour	2.410	(1.440, 4.032)	2.004	(1.064, 3.776)
In-community manual labour	1.757	(1.030, 2.996)	1.572	(0.796, 3.104)
Non-manual labour	1.563	(0.944, 2.588)	1.229	(0.640, 2.358)

*Note*: Age-stratified negative binomial model results for *P. vivax* and *P. falciparum* comparing incidence rate ratios (IRRs) and 95% confidence intervals for covariates in the migration typology within the participants aged 15 years or older. This is based on multivariate models using 1,228 observations.

**Table 5.** Negative binomial regression results for migration typology in participants under 15

	*P. vivax*		*P. falciparum*	
	IRR	95% Confidence interval	IRR	95% Confidence interval
Intercept	0.068	(0.015, 0.317)	0.059	(0.002, 2.241)
Migration status				
Non-migrant	Reference		Reference	
Low-frequency migrant	4.140	(2.244, 7.638)	5.599	(1.619, 19.354)
High-frequency migrant	9.227	(4.848, 17.561)	19.06	(5.552, 65.411)
Sex				
Female	Reference		Reference	
Male	1.938	(1.350, 2.783)	1.740	(0.992, 3.052)
Age (years)				
0–4	Reference		Reference	
5–9	1.059	(0.666, 1.683)	1.028	(0.451, 2.344)
10–14	1.763	(1.179, 2.368)	3.085	(1.603, 5.935)
SES				
Q1	Reference		Reference	
Q2	0.944	(0.482, 1.851)	0.899	(0.393, 2.057)
Q3	0.691	(0.333, 1.435)	0.397	(0.150, 1.051)
Q4	0.994	(0.507, 1.949)	0.604	(0.248, 1.469)
Q5	0.141	(0.050, 0.394)	0.345	(0.002, 2.241)

*Note*: Age-stratified negative binomial model results for *P. vivax* and *P. falciparum* comparing IRRs and 95% confidence intervals for covariates in the migration typology within the participants younger than 15 years. This is based on multivariate models using 974 observations.

The interaction between high-frequency migrants and individuals older than 49 years indicated an increase in the risk of 2.800 (95% CI: 1.135, 6.096) (Supplementary Table S3). Participants classified as high-frequency migrants older than 49 years were at nearly three times the risk of low-frequency migrants aged 15–19 years, with an IRR of 2.800 (95% CI: 1.135, 6.096). Low-frequency migrants older than 49 years were at only a tenth of the risk as low-frequency migrants aged 15–19 years, with an IRR of 0.170 (95% CI: 0.072, 0.399).

Compared to those with no education, those with a primary education, secondary education, and over a secondary education had IRRs of 0.637 (95% CI: 0.402, 1.008), 0.517 (95% CI: 0.313, 0.852), and 0.316 (95% CI: 0.152, 0.657), respectively, when restricted to participants aged 15 years and older (Table 4).

For those working in non-manual labour occupations (motorbike drives, craftsmen, merchants, businessmen), adjusted IRRs were 1.563 (95% CI: 0.944, 2.588) times greater than those not working (students and minors), these estimates were not outside of the expected range. IRRs for manual labour outside (logging and fishing) and inside of the community (agriculture, port workers) were higher than those not working in adjusted models (Table 4). High-frequency migrants employed in manual labour within their community were at an increased risk of contracting *P. vivax,* with a 2.455 (95% CI: 1.113, 5.416) times higher risk than low-frequency migrants who are not working (Supplementary Table S4). This group is also at an increased risk of 1.796 (95% CI: 0.799, 4.036) compared to low-frequency migrants employed in manual labour within the community.

The interaction generated from migrants classified as male high-frequency migrants was associated with 4.380 (95% CI: 2.968, 6.462) times greater risk than female low-frequency migrants and 2.111 (95% CI: 1.359, 3.280) times greater risk than male low-frequency migrants (Supplementary Table S5). Furthermore, female high-frequency migrants were at 3.661 (95% CI: 2.474, 5.419) greater risk than female low-frequency migrants. Male high-frequency migrants were at 1.20 (95% CI: 0.872, 1.641) times greater risk of *P. vivax* than female high-frequency migrants, though this result is not statistically significant.

Neither sex nor SES was statistically significant in models estimating the *P. vivax* incidence for participants aged 15 years and older (Table 4). The richest quintile in participants younger than 15 years was at significantly lower risk of *P. vivax* infection than the poorest quintile (Table 5). The addition of interaction terms for education, SES, age, sex, and occupation with migration status did not improve the models for *P. falciparum* infections nor did interaction terms for migration status by SES and education in models for *P. vivax* (Supplementary Tables S6 and S7).

### Adjusted estimates for Plasmodium falciparum

Adjusted IRR estimates for low-frequency migrants were 7.49 (95% CI: 1.791, 31.288) and high-frequency migrants were 33.16 (95% CI: 8.156, 134.830) times as likely to contract *P. falciparum* than non-migrants (Table 4). Although more *P. falciparum* cases were reported in women, the adjusted IRR comparing women to men was 1.965 (95% CI: 1.387, 2.786) (Table 4). While this estimate was statistically significant, IRRs for those younger than 15 years were not statistically significant (Table 5).

The adjusted IR for P. falciparum cases for individuals with more than a secondary education was 0.285 (95% CI: 0.118, 0.693) times lower than the IR of participants with less than a primary level education. IRRs for individuals with only a secondary level or only primary level education were 0.391 (95% CI: 0.222, 0.690) and 0.627 (95% CI: 0.381, 1.032) times lower compared to individuals with no education, respectively, in adjusted models (Table 4).

Study participants aged 30 years and older were at reduced risk of *P. falciparum* as compared to individuals aged 15–19 years in the adjusted model. The IRRs were 0.740 (95% CI: 0.497, 1.101), 0.669 (95% CI: 0.420, 1.036), 0.635 (95% CI: 0.402, 1.002), and 0.505 95% CI: (0.310, 0.823) for study participants aged 20–29, 30–39, 40–49 years, and older than 49 years, respectively (Table 4). Based on models including participants younger than 15 years, only participants aged 10–14 years were significant, with an IRR of 3.085 (95% CI: 1.603, 5.935), compared to participants aged 0–4 years (Table 5).

Only estimates for manual labour outside of the community, including logging and fishing, were significant. Individuals in these occupations reported an IRR of *P. falciparum* of 2.00 (95% CI: 1.064, 3.776) compared to participants not working (students and minors). Similarly, only the richest quintile (Q5) for SES was statistically significant. IRRs for those in the richest (Q1) as compared to the poorest quintile was 0.36 (95% CI: 0.205, 0.650). The IRR was 0.83 (95% CI: 0.546, 1.254) for Q2, 0.75 (95% CI: 0.480, 1.157) for Q3, and 0.89 (95% CI: 0.580, 1.372) for Q4. Household size was not related to incidence rates of malaria in the univariate models, resulting in the exclusion of the variable from the multivariate model.

Adjusted incidence rates of *P. falciparum* infection indicate higher risks of malaria infection for travel to lakes, rivers, and streams. Adjusted incidence rates were 4.341 (95% CI: 3.495, 5.372), 4.278 (95% CI: 3.476, 5.279), and 25.35 (16.178, 41.987) times higher for *P. falciparum* infection for individuals who travelled to lakes, rivers, and streams, respectively, than those who did not visit each respective destination type at any point during the study period (Table 6).

**Table 6.** Adjusted incidence rates of malaria cases by travel characteristics of migrants

	*P. vivax*			*P. falciparum*		
	*N* (%)	IR (95% CI)	IRR (95% CI)	*N* (%)	IR (95% CI)	IRR (95% CI)
Average number of seasonal trips reported						
No trips	4 (0.0)	0.668 (0.278, 1.606)	Reference	3 (0.0)	0.401 (0.129, 1.243)	Reference
1–2 trips	187 (33.6)	5.668 (5.069, 6.337)	7.216 (2.942, 17.699)	75 (24.8)	1.527 (1.232, 1.894)	3.419 (1.062, 11.009)
3–4 trips	313 (56.3)	16.879 (15.643, 18.213)	21.755 (8.883, 53.280)	179 (59.1)	6.025 (5.304, 6.843)	14.426 (4.528, 45.958)
>5 trips	52 (9.4)	18.104 (15.376, 21.316)	31.075 (12.491, 77.309)	46 (15.2)	8.675 (6.852, 10.983)	24.558 (7.535, 80.037)
Average duration of trips (days)						
1–7	299 (53.8)	12.348 (11.425, 13.346)	Reference	167 (55.1)	4.407 (3.870, 5.020)	Reference
8–14	172 (30.9)	11.650 (10.458, 12.977)	0.876 (0.752, 1.019)	85 (28.1)	3.777 (3.125, 4.565)	0.947 (0.734, 1.223)
15–21	50 (9.0)	9.806 (8.060, 11.929)	0.861 (0.682, 1.087)	31 (10.2)	3.530 (2.546, 4.894)	0.883 (0.604, 1.291)
22–30	15 (2.7)	4.598 (3.028, 6.983)	0.498 (0.315, 0.787)	11 (3.6)	2.508 (1.424, 4.416)	0.875 (0.477, 1.605)
31–60	13 (2.3)	5.513 (3.630, 8.373)	0.602 (0.369, 0.982)	6 (2.0)	1.754 (0.836, 3.680)	0.628 (0.275, 1.435)
>60	7 (1.3)	1.067 (0.591, 1.926)	0.105 (0.567, 0.196)	1 (1.0)	0.291 (0.094, 0.902)	0.078 (0.024, 0.248)
Cases by destination type^a^						
Community	529 (27.0)	11.026 (10.386, 11.705)	2.824 (5.804, 8.439)	290 (14.8)	3.873 (3.502, 4.284)	3.189 (1.877, 5.889)
Lake	127 (55.0)	25.994 (23.213, 29.108)	3.089 (2.698, 3.530)	92 (39.8)	11.524 (9.723, 13.659)	4.341 (3.495, 5.372)
River	274 (47.0)	19.902 (18.359, 21.574)	2.978 (2.644, 3.355)	185 (31.2)	8.129 (7.165, 9.223)	4.278 (3.476, 5.279)
Stream	465 (50.2)	21.525 (20.228, 22.906)	10.667 (8.86, 12.934)	283 (30.6)	8.056 (7.278, 8.917)	25.350 (16.178, 41.987)
Seasonal waterway	135 (48.7)	21.180 (18.893, 23.745)	2.439 (2.128, 14.989)	96 (34.7)	9.365 (7.886, 11.122)	3.404 (2.737, 4.217)
Rural	551 (27.2)	10.278 (9.693, 10.897)	NE	300 (14.8)	3.594 (3.255, 3.968)	NE
Urban	0 (0.0)	NE	NE	0 (0.0)	NE	NE

*Note*: *N* refers to the number of individuals who contracted malaria with the percentages out of the total number of people who travel to a particular destination type. Incidence rates were calculated for infection of malaria in the population who travelled at least one time for the average duration of trips. All cases were reported for trips to rural communities. The incidence rate ratio for trips could not be calculated for rural to urban destinations; not estimable (NE).

a For incidence calculations for different destination types, individuals may count for more than one destination. Participants may have travelled to multiple destination types throughout the follow-up period. *N* represents individuals who travelled to each destination and were diagnosed with malaria. These IRRs calculated for anyone who travelled to that destination type at least once with all individuals who never travelled to that destination as the refence group. Percentages are calculated for those who travelled to a given destination out of the total number of individuals who travelled at least once throughout the study.

## Discussion

Here, we provide prospective, population-based human cohort data to strengthen existing evidence for the effects of migration, particularly occupation-related travel, on the micro-epidemiological spread of malaria. Results support migration as a key risk factor for malaria infection, most likely due to exposure to infected mosquito populations and greater connectivity between communities that could increase transmission; however, this variable alone does not account for the increased levels of malaria reported for either species within the migrant community [9]. Other factors including destination, travel duration, occupation, education level, sex, and SES differentially influence an individual’s probability of infection.

Individuals classified as high-frequency migrants who are men or employed as manual laborers within their community were at an increased risk of malaria from *P. vivax* as compared to low-frequency travellers who were women or not working, respectively. Men far more commonly travel to bodies of water for occupational reasons [35]. This type of travel is also highly correlated with migration status. Male high-frequency migrants may be more likely to travel to more remote locations that are optimal breeding sites for the vector population and have less accessibility to prevention methods as compared to either women or low-frequency migrant subpopulations. Locations away from communities, particularly streams and rivers, may provide less access to preventative measures such as bed nets and residual insecticide spraying [36–38]. These highly mobile occupations are predominantly held by men, which differentiates the destinations visited by sex. Men had higher incidence rates of both *Plasmodium* species across all three levels of migrants than women. This trends likely results from higher exposure to mosquito habitats due to occupation-related travel and occupation type as manual labour was associated with a higher risk of malaria than non-manual labour [37].

Deforestation has been identified as a key contributor to high malaria incidence. One study conducted in the Peruvian Amazon identified *Anopheles* *darlingi* larvae in 24.1% bodies of water with forested cover compared to 41.0% of forested sites without forest cover [39]. Land clearing for farming and mining operations, creation of dams, and installing irrigation systems result in pooling of water, which generates breeding sites for malaria vector populations [39, 40]. The process of land clearing changes the level of human interaction with vector species by bringing in people to clear the land [40]. Therefore, migration still affects the incidence of malaria through attracting loggers and other relevant occupations to the area. This movement transmits the parasite from clearing sites to migrants’ home communities.

The rainy season was associated with a higher likelihood of travel than the dry season, as well as migration events associated with a high streamflow level. These associations within the cohort may increase the risk of contracting malaria based on higher *Anopheles* population density in the rainy season, which is of particular importance as a higher frequency of migration is connected to malaria infections [41]. This trend aligns with the anticipated association of higher travel in the rainy season, particularly for certain occupations that rely more on rainfall or river for travel [42–44]. However, interviews from a study conducted in Loreto show that higher river heights discourage travel upstream. Lower river heights in the dry season may even reduce travel time and increase navigability of rivers as the currents are not as strong as those during the rainy season [45, 46]. Further research is needed to investigate the level of transmission of malaria occurring within households of travellers of various frequencies, which was beyond the scope of this study. Additionally, the distance travelled by migrants should be investigated to further understand the implications of time spent moving throughout the river system. A comparison of the different communities, streams, and rivers may also provide insights into levels of transmission at particular locations.

These data were collected between 2006 and 2009; however, the malaria context is similar to current conditions in Loreto. While the malaria incidence declined significantly from 2006 to 2010 in Peru due to the PAMAFRO program [47], incidence rates have rebounded since 2011. In Loreto, there has been no new road construction since 2009, which could have altered migration patterns. Moreover, malaria seasonality patterns remain consistent today compared to those in the 2006–2009 period.

### Limitations

Data on the purpose of travel were not collected, limiting our understanding of the extent to which river height may impact the timing of an individual’s trips. Some travel requires certain thresholds for river heights, such as the lumber industry, which needs the rivers to be high enough to float logs downstream, while other forms of travel, such as children going to school, do not depend on river height. If these data were present, analyses could be stratified by the presence of these requirements, allowing us to understand trends in the cases where river height matters.

Censoring the data by six months may bias against very high-frequency travellers who spend large amounts of time outside of the community. However, it allows for a minimum of three data points from malaria tests for each individual. Similarly, within this analysis, potential for misclassification exists in case censoring based on the 28-day assumptions of recurrent cases. Individuals with multiple infections within this window may have resulted from two unique infections, and recurrence is possible after the window specified.

Covariates regarding malaria prevention methods were not accounted for in this analysis as these data were not collected throughout the study, restricting evidence for the temporal relationship to malaria infection. These data also do not exist for the destinations of travel. The level of access to and use of preventative measures when away from home are unknown.

## Supporting information

Gunderson et al. supplementary material 1 Gunderson et al. supplementary material

Gunderson et al. supplementary material 2 Gunderson et al. supplementary material

## Supplementary material

The supplementary material for this article can be found at https://doi.org/10.1017/S0950268823001838.
