# A prospective cohort study linking migration, climate, and malaria risk in the Peruvian Amazon – CORRIGENDUM

**DOI:** 10.1017/S0950268824000025

**Published:** 2024-01-12

**Authors:** Annika K. Gunderson, Cristina Recalde-Coronel, Benjamin F. Zaitchik, Pablo Peñataro Yori, Silvia Rengifo Pinedo, Maribel Paredes Olortegui, Margaret Kosek, Joseph M. Vinetz, William K. Pan

The original version of this article contained an incorrectly spelled author name.

The correct spelling is ‘Benjamin F. Zaitchik’.
