# Supplementary figures and images for: A prospective cohort study linking migration, climate, and malaria risk in the Peruvian Amazon
Source: Epidemiol Infect. 2023 Nov 30;151:e202. doi: 10.1017/S0950268823001838 (PMC10753477; doi:10.1017/S0950268823001838)

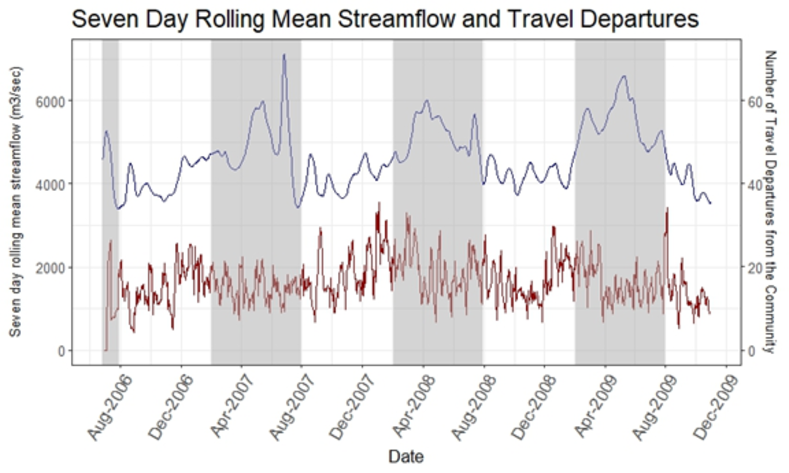

Supplement: Gunderson et al. supplementary material 1 — Gunderson et al. supplementary material [file S0950268823001838sup001.png]
