# Supplementary material for: A prospective cohort study linking migration, climate, and malaria risk in the Peruvian Amazon
Source: Epidemiol Infect. 2023 Nov 30;151:e202. doi: 10.1017/S0950268823001838 (PMC10753477; doi:10.1017/S0950268823001838)
Supplement: Gunderson et al. supplementary material 2 — Gunderson et al. supplementary material [file S0950268823001838sup002.docx]

**Supplemental Figures and Tables**

Figure S1. *Seven Day Rolling Mean Streamflow and Travel Departures*. The blue line represents the seven-day rolling mean (7DRM) streamflow (m^3^/sec) for the selected region of the Napo River running along Mazan for the duration of the study. The red line represents the total number of travel departures on a given day by members of the cohort. Shaded regions indicate the rainy season.

Table S1. *Negative Binomial Regression Results for Migration Typology in Participants Over 15* Age-stratified negative binomial model results for *P. vivax* and *P. falciparum* comparing incidence rate ratios (IRRs) and 95% confidence intervals for covariates in the migration typology within the participants 15 years or older in age. This is based on multivariate models using 1228 observations.

| Table S1. Negative Binomial Regression Results for Migration Typology in Participants Over 15 | | | | |
| --- | --- | --- | --- | --- |
|  | *P. vivax* | | *P. falciparum* | |
|  | IRR | 95% Confidence Interval | IRR | 95% Confidence Interval |
| Intercept | 0.131 | (0.053, 0.322) | 2.556 | (2.40e-13, 2.72e+13) |
| Migration Status | | | | |
| Non-Migrant | Reference |  | Reference |  |
| Low Frequency Migrant | 2.929 | (1.660, 5.170) | 7.486 | (1.791, 31.288) |
| High Frequency Migrant | 7.749 | (4.473, 13.424) | 33.161 | (8.156, 134.830) |
| Sex | | | | |
| Female | Reference |  | Reference |  |
| Male | 1.430 | (1.093, 1.872) | 1.965 | (1.387, 2.786) |
| Age | | | | |
| 15-19 years | Reference |  | Reference |  |
| 20-29 years | 0.640 | (0.456, 0.899) | 0.740 | (0.497, 1.101) |
| 30-39 years | 0.504 | (0.345, 0.737) | 0.669 | (0.420, 1.036) |
| 40-49 years | 0.444 | (0.296, 0.667) | 0.635 | (0.402, 1.002) |
| >49 years | 0.391 | (0.253, 0.605) | 0.505 | (0.310, 0.823) |
| Education level | | | | |
| No education | Reference |  | Reference |  |
| Primary education | 0.637 | (0.402, 1.008) | 0.627 | (0.381, 1.032) |
| Secondary education | 0.517 | (0.313, 0.852) | 0.391 | (0.222, 0.690) |
| >Secondary education | 0.316 | (0.152, 0.657) | 0.285 | (0.118, 0.693) |
| SES | | | | |
| Q1 | Reference |  | Reference |  |
| Q2 | 0.970 | (0.682, 1.380) | 0.828 | (0.546, 1.254) |
| Q3 | 0.996 | (0.697, 1.423) | 0.745 | (0.480, 1.157) |
| Q4 | 1.307 | (0.922, 1.852) | 0.892 | (0.580, 1.372) |
| Q5 | 0.724 | (0.473, 1.109) | 0.365 | (0.205, 0.649) |
| Occupation | | | | |
| No work | Reference |  | Reference |  |
| Out of community manual labour | 2.410 | (1.440, 4.032) | 2.004 | (1.064, 3.776) |
| In community manual labour | 1.757 | (1.030, 2.996) | 1.572 | (0.796, 3.104) |
| Not manual labour | 1.563 | (0.944, 2.588) | 1.229 | (0.640, 2.358) |

Table S2. *Negative Binomial Regression Results for Migration Typology in Participants Under 15* Age-stratified negative binomial model results for *P. vivax* and *P. falciparum* comparing IRRs and 95% confidence intervals for covariates in the migration typology within the participants under 15 years old. This is based on multivariate models using 974 observations.

| Table S2. Negative Binomial Regression Results for Migration Typology in Participants Under 15 | | | | |
| --- | --- | --- | --- | --- |
|  | *P. vivax* | | *P. falciparum* | |
|  | IRR | 95% Confidence Interval | IRR | 95% Confidence Interval |
| Intercept | 0.068 | (0.015, 0.317) | 0.059 | (0.002, 2.241) |
| Migration Status | | | | |
| Non-Migrant | Reference |  | Reference |  |
| Low Frequency Migrant | 4.140 | (2.244, 7.638) | 5.599 | (1.619, 19.354) |
| High Frequency Migrant | 9.227 | (4.848, 17.561) | 19.06 | (5.552, 65.411) |
| Sex | | | | |
| Female | Reference |  | Reference |  |
| Male | 1.938 | (1.350, 2.783) | 1.740 | (0.992, 3.052) |
| Age | | | | |
| 0-4 years | Reference |  | Reference |  |
| 5-9 years | 1.059 | (0.666, 1.683) | 1.028 | (0.451, 2.344) |
| 10-14 years | 1.763 | (1.179, 2.368) | 3.085 | (1.603, 5.935) |
| SES | | | | |
| Q1 | Reference |  | Reference |  |
| Q2 | 0.944 | (0.482, 1.851) | 0.899 | (0.393, 2.057) |
| Q3 | 0.691 | (0.333, 1.435) | 0.397 | (0.150, 1.051) |
| Q4 | 0.994 | (0.507, 1.949) | 0.604 | (0.248, 1.469) |
| Q5 | 0.141 | (0.050, 0.394) | 0.345 | (0.002, 2.241) |

Table S3. *Negative Binomial Regression Results for Interaction of Migration Category and Age* Negative binomial model results for *P. vivax* and *P. falciparum* comparing IRRs and 95% confidence intervals for covariates in the migration typology within the participants 15 years or older in age. This is based on multivariate models using 1228 observations. Models assessing *P. vivax* also controlled for SES, sex, education, occupation, and marital status. Models assessing *P. falciparum* also controlled for SES, sex, education, occupation, and relationship in the family (i.e., daughter, father).

| Table S3. Negative Binomial Regression Results for Interaction of Migration Category and Age | | | | |
| --- | --- | --- | --- | --- |
|  | *P. vivax* | | *P. falciparum* | |
|  | IRRs | 95% Confidence Interval | IRRs | 95% Confidence Interval |
| Intercept | 0.490 | (0.211, 1.140) | 45093.63 | (0, .) |
| Migration Status | | | | |
| Low Frequency Migrant | Reference |  | Reference |  |
| High Frequency Migrant | 2.098 | (1.229, 3.579) | 3.935 | (1.712, 9.047) |
| Age | | | | |
| 15-19 years | Reference |  | Reference |  |
| 20-29 years | 0.494 | (0.261, 0.935) | 0.715 | (0.547, 1.254) |
| 30-39 years | 0.558 | (0.286, 1.087) | 0.443 | (0.477, 1.146) |
| 40-49 years | 0.367 | (0.170, 0.792) | 0.919 | (0.577, 1.354) |
| >49 years | 0.170 | (0.072, 0.399) | 0.247 | (0.211, 0.667) |
| Migration and Age Interaction | | | | |
| Low Frequency Migrant & 15-19 years | Reference |  | Reference |  |
| High Frequency Migrant & 20-29 years | 1.324 | (0.666, 2.632) | 2.906 | (1.240, 6.809) |
| High Frequency Migrant & 30-39 years | 0.773 | (0.373, 1.601) | 2.672 | (1.112, 6.423) |
| High Frequency Migrant & 40-49 years | 1.309 | (0.571, 2.999) | 2.293 | (0.947, 5.549) |
| High Frequency Migrant & >49 years | 2.800 | (1.135, 6.096) | 2.159 | (0.882, 5.287) |

Table S4. *Negative Binomial Regression Results for Interaction of Migration Category and Occupation* Negative binomial model results for *P. vivax* and *P. falciparum* comparing IRRs and 95% confidence intervals for covariates in the migration typology within the participants 15 years or older in age. This is based on multivariate models using 1228 observations. Models assessing *P. vivax* also controlled for SES, sex, education, age, and marital status. Models assessing *P. falciparum* also controlled for SES, sex, education, age, and relationship in the family (i.e., daughter, father).

| Table S4. Negative Binomial Regression Results for Interaction of Migration Category and Occupation | | | | |
| --- | --- | --- | --- | --- |
|  | *P. vivax* | | *P. falciparum* | |
|  | IRR | 95% Confidence Interval | IRR | 95% Confidence Interval |
| Intercept | 0.399 | (0.157, 1.013) | 13.437 | (6.26e-14, 2.88e15) |
| Migration Status | | | | |
| Low Frequency Migrant | Reference |  | Reference |  |
| High Frequency Migrant | 2.968 | (1.314, 6.705) | 7.401 | (1.627,33.674) |
| Occupation | | | | |
| No work | Reference |  | Reference |  |
| Out of community manual labour | 4.349 | (1.856, 10.188) | 2.645 | (0.480, 14.556) |
| In community manual labour | 1.838 | (0.818, 4.129) | 2.343 | (0.495, 11.087) |
| Not manual labour | 1.240 | (0.562, 2.736) | 2.213 | (0.486, 10.887) |
| Migration and Occupation Interaction | | | | |
| Low Frequency Migrant & No work | Reference |  | Reference |  |
| High Frequency Migrant & Out of community manual labour | 2.455 | (1.113, 5.416) | 13.892 | (3.242, 59.527) |
| High Frequency Migrant & In community manual labour | 1.796 | (0.799, 4.036) | 11.097 | (2.527, 48.731) |
| High Frequency Migrant & Not manual labour | 1.874 | (0.886, 3.966) | 7.957 | (1.853, 34.161) |

Table S5. *Negative Binomial Regression Results for Interaction of Migration Category and Sex* Negative binomial model results for *P. vivax* and *P. falciparum* comparing IRR and 95% confidence intervals for covariates in the migration typology within the participants 15 years or older in age. This is based on multivariate models using 1228 observations. Models assessing *P. vivax* also controlled for education, SES, age, occupation, and marital status. Models assessing *P. falciparum* also controlled for SES, age, education, occupation, and relationship in the family (i.e., daughter, father).

| Table S5. Negative Binomial Regression Results for Interaction of Migration Category and Sex | | | | |
| --- | --- | --- | --- | --- |
|  | *P. vivax* | | *P. falciparum* | |
|  | IRR | 95% Confidence Interval | IRR | 95% Confidence Interval |
| Intercept | 0.357 | (0.159, 0.801) | 11.452 | (1.32e-06, 1.01e08) |
| Migration Status | | | | |
| Low Frequency Migrant | Reference |  | Reference |  |
| High Frequency Migrant | 3.661 | (2.474, 5.419) | 3.602 | (2.106, 6.162) |
| Sex | | | | |
| Female | Reference |  | Reference |  |
| Male | 2.111 | (1.359, 3.280) | 1.480 | (0.754, 2.904) |
| Migration and Sex Interaction | | | | |
| Low Frequency Migrant & Female | Reference |  | Reference |  |
| High Frequency Migrant & Male | 4.380 | (2.968, 6.462) | 7.742 | (4.625, 12.961) |

Table S6. *Negative Binomial Regression Results for Interaction of Migration Category and SES* Negative binomial model results for *P. vivax* and *P. falciparum* comparing IRR and 95% confidence intervals for covariates in the migration typology within the participants 15 years or older in age. This is based on multivariate models using 1228 observations. Models assessing *P. vivax* also controlled for education, sex, age, occupation, and marital status. Models assessing *P. falciparum* also controlled for age, sex, education, occupation, and relationship in the family (i.e., daughter, father).

| Table S6. Negative Binomial Regression Results for Interaction of Migration Category and SES | | | | |
| --- | --- | --- | --- | --- |
|  | *P. vivax* | | *P. falciparum* | |
|  | IRR | 95% Confidence Interval | IRR | 95% Confidence Interval |
| Intercept | 0.462 | (0.198, 1.076) | 14009.02 | (0, .) |
| Migration Status | | | | |
| Low Frequency Migrant | Reference |  | Reference |  |
| High Frequency Migrant | 2.389 | (1.418, 4.025) | 3.399 | (1.793, 6.444) |
| SES | | | | |
| Q1 | Reference |  | Reference |  |
| Q2 | 0.887 | (0.468, 1.682) | 0.601 | (0.230, 1.569) |
| Q3 | 0.963 | (0.516, 1.797) | 0.516 | (0.198, 1.345) |
| Q4 | 0.825 | (0.417, 1.636) | 0.770 | (0.313, 1.898) |
| Q5 | 0.854 | (0.413, 1.767) | 0.213 | (0.046, 0.979) |
| Migration and SES Interaction | | | | |
| Low Frequency Migrant & Q1 | Reference |  | Reference |  |
| High Frequency Migrant & Q2 | 2.298 | (1.348, 3.918) | 3.064 | (1.577, 5.956) |
| High Frequency Migrant & Q3 | 2.354 | (1.367, 4.053) | 2.806 | (1.412, 5.575) |
| High Frequency Migrant & Q4 | 3.337 | (1.981, 5.622) | 3.137 | (1.597, 6.164) |
| High Frequency Migrant & Q5 | 1.582 | (0.871, 2.875) | 1.392 | (0.636, 3.048) |

Table S7. *Negative Binomial Regression Results for Interaction of Migration Category and Education* Negative binomial model results for *P. vivax* and *P. falciparum* comparing IRR and 95% confidence intervals for covariates in the migration typology within the participants 15 years or older in age. This is based on multivariate models using 1228 observations. Models assessing *P. vivax* also controlled for SES, sex, age, occupation, and marital status. Models assessing *P. falciparum* also controlled for SES, sex, age, occupation, and relationship in the family (i.e., daughter, father).

| Table S7. Negative Binomial Regression Results for Interaction of Migration Category and Education | | | | |
| --- | --- | --- | --- | --- |
|  | *P. vivax* | | *P. falciparum* | |
|  | IRR | 95% Confidence Interval | IRR | 95% Confidence Interval |
| Intercept | 0.228 | (0.061, 0.851) | 5.442 | (7.31e-10, 4.05e10) |
| Migration Status | | | | |
| Low Frequency Migrant | Reference |  | Reference |  |
| High Frequency Migrant | 5.606 | (1.635, 19.222) | 11.981 | (1.564, 91.760) |
| Education | | | | |
| No education | Reference |  | Reference |  |
| Primary education | 1.354 | (0.415, 4.423) | 1.763 | (0.231, 13.475) |
| Secondary education | 0.759 | (0.221, 2.600) | 0.992 | (0.120, 8.224) |
| >Secondary education | 0.394 | (0.077, 2.023) | 0.530 | (0.032, 8.883) |
| Migration and Education Interaction | | | | |
| Low Frequency Migrant & No education | Reference |  | Reference |  |
| High Frequency Migrant & Primary education | 2.855 | (0.881, 9.253) | 7.037 | (0.943, 52.507) |
| High Frequency Migrant & Secondary education | 2.472 | (0.751, 8.139) | 4.520 | (0.593, 34.476) |
| High Frequency Migrant & >Secondary education | 1.840 | (0.488, 6.929) | 3.494 | (0.405, 30.100) |
